# Strumal Carcinoid Tumor of the Ovary: Report of Rare Occurrence with Review of Literature

**DOI:** 10.3390/diagnostics12112706

**Published:** 2022-11-05

**Authors:** Li-Ping Shen, An-Qiang Yang, Lei Jin

**Affiliations:** 1 Department of Gynecology, Changning Maternity and Infant Health Hospital, East China Normal University, Shanghai 200065, China; 2 Department of Pathology, Changning Maternity and Infant Health Hospital, East China Normal University, Shanghai 200065, China; 3 Department of Rheumatology and Immunology, Tongren Hospital, Shanghai Jiao Tong University School of Medicine, Shanghai 200336, China

**Keywords:** strumal carcinoid tumor of the ovary, ovarian carcinoid tumor, diagnosis

## Abstract

The primary ovarian carcinoid tumor is a very rare ovarian tumor, which accounts for approximately 0.5% to 1.7% of all carcinoids and 1% of ovarian cancer. According to its histopathological features, it can be divided into four categories: insular, trabecular, strumal, and mucinous, among which insular carcinoid is common in Western countries. By comparison, the chain-typed and trabecular carcinoid seem to be common in Asian countries. To date, about 150 cases have been reported in the world, and 40% of them are strumal carcinoid tumor of the ovary (SCTO), which is a highly specialized teratoma differentiated from the monomer, and often characterized by the coexistence of thyroid follicular tissue and carcinoid tissue with the neuroendocrine function. Preoperative diagnosis may be difficult due to the very insidious nature of the disease and its multiple imaging manifestations. We reported the case of a 39-year-old woman with a 5-year clinical history. Gynecologic examination and ultrasonic testing revealed an enlarged ovary with a diameter of about 60 mm, accompanied by a hypoechoic area, which was suspected to be a benign teratoma. Ca-125, AFP, free T4, TSH, and other diagnostic indicators were normal. During the laparoscopic oophorocystectomy of the left ovary, a smooth and solid tumor with the size of 6 × 6 × 5 cm was found in the right ovary. During the operation, a mature cystic teratoma containing a struma was frozen, then the oophorocystectomy of the left ovary was performed. According to the Federation International of Gynecology and Obstetrics (FIGO) in 2014, histopathological examination showed a mature teratoma with thyroid carcinoid stage Ic, and Douglas’s cystic hygroma cytopathology was negative. One year after the operation, the patient was tumor-free, with Ca-125, FT4, and TSH being within the normal range. Specific diagnostic tools and serological monitoring of malignant tumors of the ovary have low specificity and sensitivity in the diagnosis of this rare malignant tumor of the ovary. Female patients with habitual constipation, chronic abdominal colic, diarrhea, and endocrine dysfunction also need to be alert to this rare malignant tumor of the ovary.

## 1. Background

Strumal carcinoid tumor of the ovary (SCTO) is a rare and unique teratoma of the ovary, which consists of thyroid tissue mixed with neuroendocrine neoplasm (carcinoid). To date, about 150 cases of primary ovarian carcinoid have been reported in the world, and 40% of them are strumal carcinoid tumor of the ovary (SCTO) [1]. As there are no known reviews or case series discussing SOCT independent of carcinoid tumors arising in association with other neoplasms, the true incidence remains unknown [2]. In 2014, the World Health Organization (WHO) classified the tumor as a monoderm teratoma in its new category, believing that both components of tumors were derived from the endoderm of teratomas [3]. The age of SCTO onset is mostly from 21 to 77 years old, with an average age of 53 years old [4]. Most SCTO cases are found in women who are in the perimenopausal or postmenopausal period. Usually, it is unilateral and may exist alone or occur in combination with teratomas, mucinous cystadenoma, and Brenner tumors, and it can also be accompanied by contralateral mature cystic teratomas or mucinous cystadenomas. When occurring in bilateral ovaries, it is mostly the metastatic ovarian carcinoid [5].

## 2. Case Presentation

**Figure 1:**
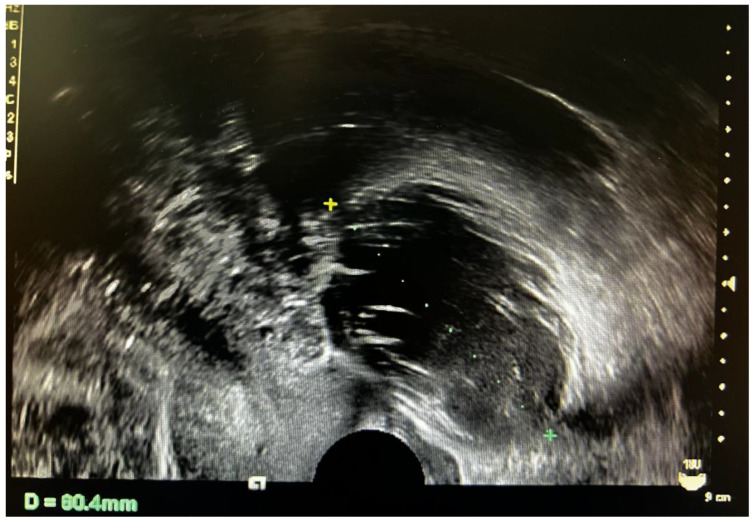
Ultrasound revealed a mixed echogenic zone on the left side of the uterus, 55 × 60 × 46 mm, with a poorly defined boundary, medium hyperechogenic mass in the inner part, 21 × 12 × 18 mm, and sparse color blood flow on the surface and inside.

**Figure 2:**
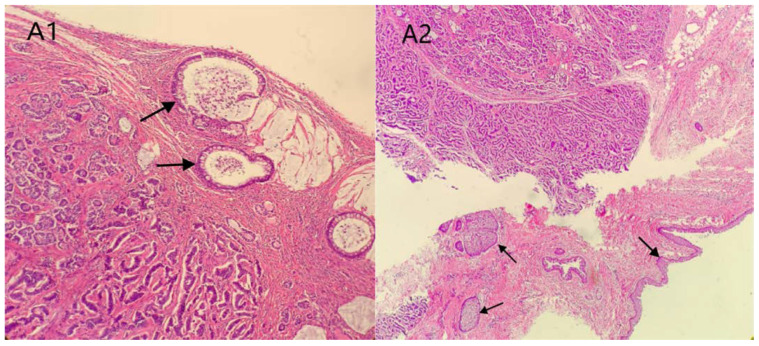

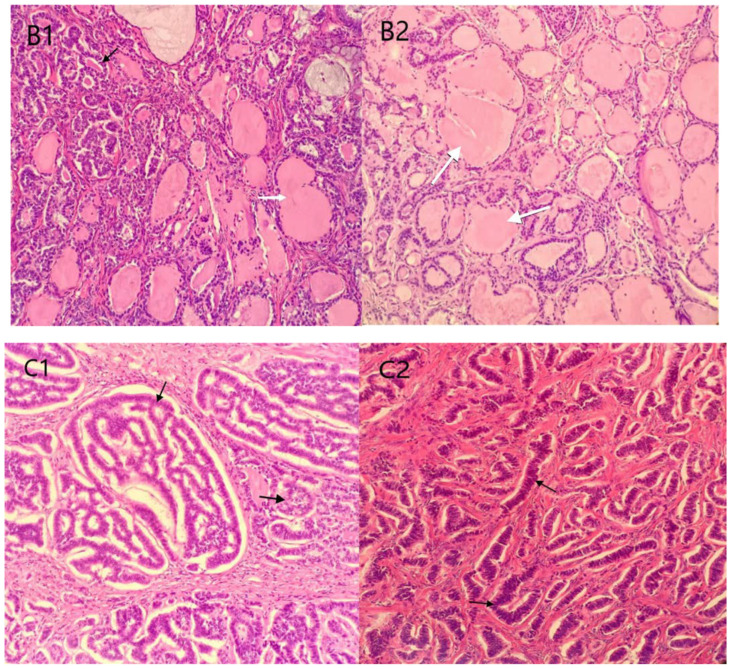
Mature cystic teratoma: stratified squamous epithelium with skin appendages, matureneuronal elements and fat tissue; (**A1**): Mature cystic teratoma (colonic gland), H&E × 100; (**A2**): Mature cystic teratoma (skin and cutaneous appendages), H&E × 40. (**B1**): carcinoid components: cancer cells are arranged in trabecular, ribbon, and adenoid patterns, locally nested or insular, tumor cells are uniform and consistent, cytoplasm is abundant and eosinophilic, nuclei are round and centered, nuclear chromatin is uniform and coarse particles, and mitotic figures are rare can be seen in the tumor parenchyma (black arrow). (**B2**): strumal components: thyroid follicles of different sizes, follicles lined by a single layer of flat epithelium or cuboidal epithelium, follicles containing eosinophilic material (white arrow). (**C1**): carcinoid components (glandular, nested, island-like structure), H&E × 200. (**C2**): carcinoid components (small bright, cable shape, ribbon-like structure), H&E × 200.

**Figure 3:**
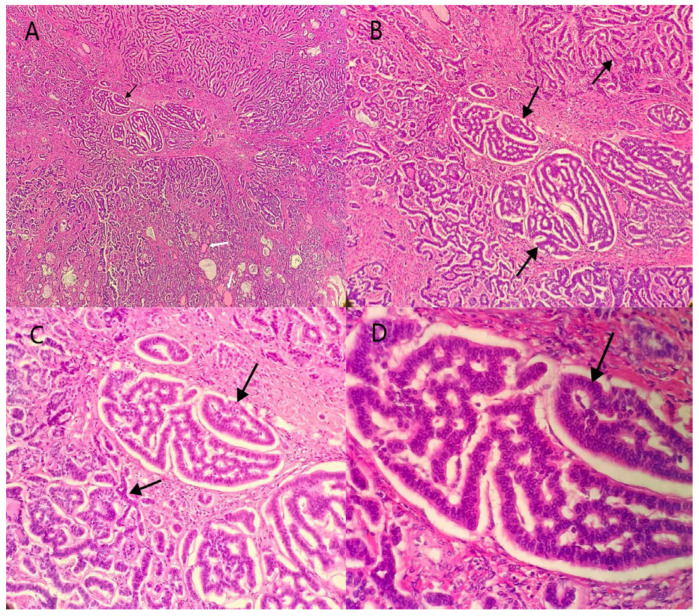
Strumal components: thyroid follicles of different sizes (white arrow);Hematoxylin-eosin staining of the strumal carcinoid tumor. The strumal component can be seen in the tumor parenchyma (black arrow). (**A**): H&E × 40; (**B**): H&E × 100; (**C**): H&E × 200; (**D**): H&E × 400.

**Figure 4:**
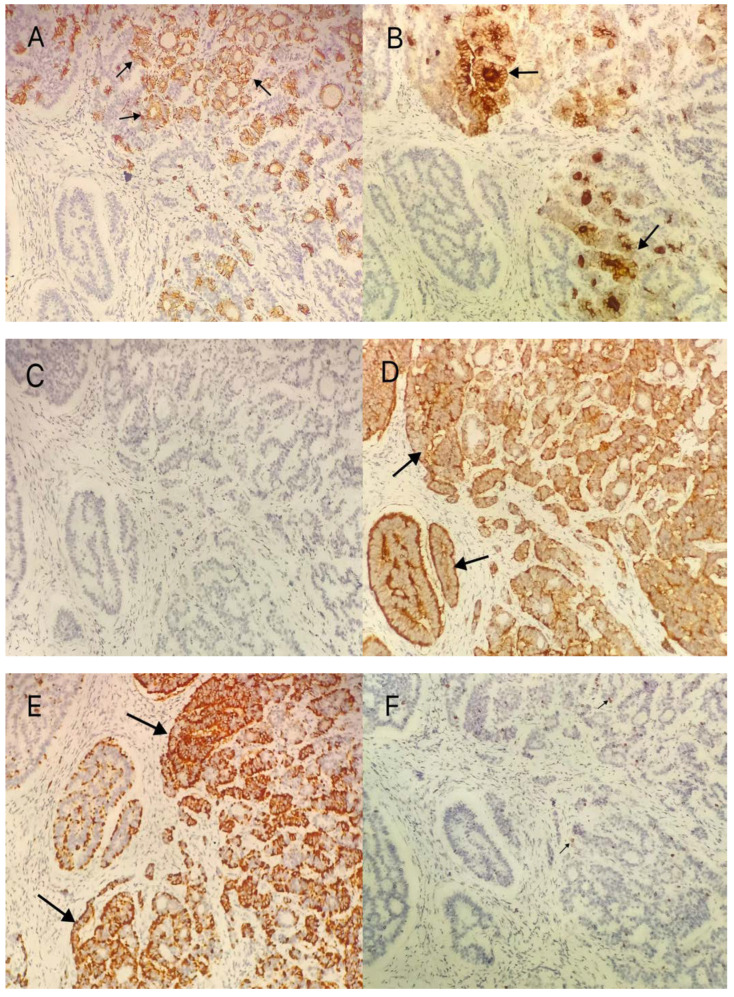
Immunohistochemically tumor cells were positive for: (**A**): CK7 (+), IHC ×200; (**B**): Tg (+), IHC × 200, (**C**): CK20 (−) IHC × 200; (**D**): Syn (++), IHC × 200; (**E**): GgA (++); IHC × 200; (**F**): Ki-67 (5%+) IHC × 200. (The black arrow indicates the site of the tumor with positive staining).

The patient was a 39-year-old woman, who was married (G1P1) and had suffered from irregular menstruation for 5–6/30–90 days. Due to the main complaint of “founding ovarian mass in physical examination 5 years ago”, she was admitted to the Gynecology Department of our hospital on 3 June 2021. In the physical examination 5 years ago, a suspected left ovarian cyst was founded with a diameter of 5 cm. After admission, Ultrasound showed a mixed echo area of 55 × 60 × 46 mm on the left side of the uterus with a poorly defined boundary, as well as a dough medium-high echo at the internal part with a size of 21 × 12 × 18 mm, meanwhile, sparse color blood flow was exhibited on the surface and inside (Figure 1). A hypoechoic area (44 × 21 × 21 mm) was seen beside the right ovary, with an irregular shape, thin wall, and sparse color blood flow on the surface. On gynecologic examination, a mass with a diameter of 6 cm was palpable in the left side of the adnexa area, with poor mobility, moderate texture, clear boundary, smooth surface, and absence of tenderness. Sex hormones: FSH: 7.21 IU/L; LH: 7.87 IU/L; PRL: 652.77 mIU/L; E2: 238.26 pmol/L; T: 1.07 nmol/L. Thyroid hormones: TPOAb: 0.50 Iu/mL; FT3: 4.52 pmol/L; FT4: 11.87 pmol/L; T3: 1.15 nmol/L; TH: 107.66 nmol/L; TSH: 1.65 mIU/L. Tumor indicators: CA125: 11.2 U/mL; CA199: 2.6 U/mL; CA153: 4.0 U/mL; AFP: 2.5 ng/mL; CEA: 1.2 ng/mL. Preoperative diagnosis: A left ovarian teratoma was suspected. Later, elective laparoscopic oophorocystectomy of the left ovary was performed. During the operation, the cystic enlargement of the left ovarian was 6 × 6 × 5 cm in size and smooth in surface, containing grease and hair. The right oviduct increased by 2 × 2 cm with no abnormality in the appearance. No obvious ascites was observed in the abdominal cavity. Intraoperative freezing: (Left ovarian cyst) Mature cystic teratoma (containing a struma, with some glands having a certain degree of atypia and a few glands interspersed in the mesenchyme) (Figure 2 and Figure 3). In consideration of the age of the patient and intraoperative findings, the laparoscopic oophorocystectomy of the left ovary was tentatively performed. Postoperative pathology: (Left ovary) Microscopic morphology combined with immunohistochemical results showed that it was consistent with mature cystic teratoma with goitrous carcinoid. Immunohistochemistry of tumor tissue: CK7 (+), Tg (+), CK20 (−), Syn (++), CgA (++), Ki-67 (5%+) (Figure 4). Postoperative diagnosis: Left ovarian mature teratoma with goitrous carcinoid. The patient was closely followed up at 3 months, 6 months, and 1 year after operation. Ultrasound showed that bilateral ovaries were normal in size and no abnormal echo area was found; no abnormality was found in tumor indicators, thyroid hormones, and sex hormones. The patient was pregnant for more than 2 months and in normal pregnancy.

## 3. Discussion and Review of Literature

A literature review of original articles, review articles, and case reports was conducted using Pubmed, Embase, and Cochrane databases around ovarian strumal carcinoid tumors. The search terms were: Ovarian strumal carcinoid; strumal carcinoid of the ovary; strumal carcinoid case report; and strumal carcinoid tumor of the ovary. We searched for studies of ovarian strumal carcinoid tumors from 2012 to 2022 and retrospectively analyzed a total of 10 studies (Table 1), including one review of teratoma and one review of carcinoid tumors at various sites, which are not included. We searched for the most recent case reports within 2017 to 2022 for a total of 12 individuals (Table 2). We performed a pooled analysis of cases based on a literature review and including the cases in this article to obtain information about the clinicopathological features, treatment strategies, and prognostic symptomatic factors of this rare disease. On this basis, we further highlighted possible relevant indicators of this disease.

### 3.1. Clinical Characteristics

No typical clinical symptoms will be manifested in SCTO, which is mostly associated with abdominal bulging and abdominal pain, as well as those signs and symptoms associated with hormonal overproduction. It has also been found incidentally through ultrasound examination [2]. In 2022, Turla A et al. [12] searched 66 original articles, collected data from 117 patients, researched the latest clinical characteristics, and found 88 symptomatic patients, 37% of whom presented with abdominal bulging, 49% with pain due to an enlarged abdominal tumor mass, and 37% due to constipation (only nine of these patients had YY peptide analysis and the results were outside the physiological range). Not only that, we can also see different clinical features in a total of 13 case reports in the last 5 years (one case in this paper and 12 cases in the literature review), such as the diagnosis of SCTO due to abdominal pain, abnormal uterine bleeding, urinary frequency, and lower limb edema. Some patients with SCTO (predominantly strumal and trabecular types) mainly present with chronic constipation, and the release of YY peptide [22] has been suggested by many authors as a possible cause of constipation symptoms in SCTO patients. In 2015, Erdenebaatar C et al. [8] reviewed 20 cases of YY positive ovarian carcinoid patients and found that Strumal and trabecular carcinoids were indeed closely related to the production of YY peptides and may cause constipation. YY peptide cannot only inhibit multiple intestinal functions, but also inhibit the movement of the jejunum and colon, having a strong inhibitory effect on peristalsis. In addition, some patients with SCTO have endocrine dysfunction, such as episodes of hypertension, hyperglycemia, or hypoglycemia. Robboy et al. [4]. summarized 50 patients with SCTO, 8% of which showed clinical symptoms of steroid hormone secretion, such as endometrial hyperplasia, hirsutism, male gonad hyperplasia, etc. The thyroid follicles in their tissues also showed thyroid function. Some patients with SCTO show typical signs and symptoms of carcinoid syndrome, such as intermittent flushing, abdominal colic, diarrhea, carcinoid heart disease, etc., which are mediated by bioactive substances produced by carcinoid cells [23]. In this case, the patients denied any history of significant abdominal pain, but it was found by pathological confirmation after the elective operation of a pelvic mass for many years. After the operation, by asking about the medical history, we found that the patient had been diagnosed with polycystic ovarian syndrome (PCOS) due to oligomenorrhea and crinosity. They stopped taking the drug after oral administration of Diane-35 (Ethinylestradiol and Cyproterone Acetate Tablets) for 2 years, then the pelvic mass was found through physical examination. We know that a few patients with SCTO may have endocrine dysfunction, which is considered to be caused by abnormal hormone secretion due to stimulation of some ovarian tissues near the tumor. 

### 3.2. Imaging Characteristics

In the imaging examination, SCTO lacks typical characteristics. The tumors are often large in size, with a statistical average diameter of 8 cm or more. It is usually manifested as unilateral cystic or solid-cystic mixed echo, which is often similar to the malignant tumor. In order to distinguish benign and malignant lesions, applying the International Ovarian Tumor Analysis (IOTA) Simple Rules (SR), the IOTA Simple Rules risk assessment (SRR), the IOTA Assessment of Different NEoplasias in the adneXa (ADNEX) model, and the Ovarian-Adnexal Reporting and Data System (O-RADS), [24] which is considered to be a valid diagnostic tool for pre-operative assessment of ovarian masses in clinical practice, especially useful for borderline ovarian tumors. The serum CA-125 level of this patient was 11.2 U/mL. Ultrasound showed that one cyst was found in the adnexal mass, with no nipple protrusion, no acoustic shadow, and a small amount of effusion in the pelvic cavity. The largest diameter of the lesion and the material part was 60 mm and 21 mm, respectively. The probabilities provided by the ADNEX model [25] were as follows: The incidence rate of benign tumors, malignant tumors, and borderline tumors was 64.0%, 36.0%, and 20.4%, respectively. Besides, the incidence of stage I cancer, stage II-IV cancer, and secondary metastatic cancer, was 7.0%, 3.3%, and 5.3%, respectively. Therefore, in this case, the possibility of the malignant tumor should not be excluded before the operation, and the operation plan should be carefully drawn up after a full evaluation and family conversation.

### 3.3. Pathological Characteristics

Primary carcinoid tumors of the ovary are an extremely rare group of diseases. Zhu R et al. [10] collected the clinicopathological data of 17 cases of primary carcinoid tumor of the ovary occurring in Peking Union Medical College Hospital from 2002 to 2017, and found that the disease is rare, commonly occurs in unilateral ovary, with nonspecific clinical features, mostly mixed with teratoma, and occasionally combined with mucinous cystadenoma, etc. In the latest 5-year case series, eight out of 13 patients, together with one case in this paper, had combined ovarian mature teratomas (one lesion in both ovaries). As can be seen, the composition of the tumor varies. When the tumor is combined with a mature teratoma, the general morphology can be typical of sebaceous, hairy, and cephalic nodes, or it can be a cystic nodule or even a completely solid nodule.

Ovarian carcinoid tumors are categorized into four groups according to the histopathological patterns: insular, trabecular, strumal, and mucinous [26]. Thereinto, trabecular, a mixture of trabecular and insular types are common [12]. Islands of uniform neoplastic cells are typical for insular carcinoids. Trabecular carcinoids are characterized by the growth of tumor cells in trabeculae and are only rarely present with endocrine manifestations [23]. Mucinous carcinoid tumors of the ovary are considered a specific histopathologic entity. Unlike other types of primary ovarian carcinoid tumors, it behaves like an aggressive malignant neoplasm [27]. The postoperative pathology of neoplasia and immunohistochemistry were the gold standard for the diagnosis of SCTO. The microscopic characteristics of strumal carcinoid are that strumal tissue and carcinoid tissue can be mixed together to varying degrees, and migration can be seen between them. Neuroendocrine cells gradually infiltrate thyroid tissue and replace follicular epithelial cells. The tumor can be seen to be composed of carcinoids, thyroid follicles, and other components. Carcinoid cytoplasm is abundant, eosinophilic, and granular, whose nuclear small chromatin is uniform and nuclear fission is rare. Thyroid follicles were mostly of normal follicular structure, lined with pavement, cubical or cylindrical epithelium, and there might be a gelatinous substance in the cavity.

Of the 13 cases reported (Table 2), there was three cases of trabecular, two cases of insular, three cases of strumal, three cases of mixed trabecular andinsular (including one in our case), one case of mixed trabecular and mucinous, and one case of unknown carcinoid type. In addition, there was one case of strumal combined with appendiceal carcinoid. In this case, teratomas were found at the periphery of the tumor, including skin and adnexa, cartilage, and colonic glands (Figure 2A1,A2); Strumal (thyroid follicles of different sizes lined with a single-layer pavement epithelium or cubical epithelium; and the follicles contained eosinophils) (Figure 2B1,B2) and carcinoid (cancer cells were arranged in a trabecular, ribbon-like, and glandular manner, and locally nested or insular; the tumor cells were uniform, and the cytoplasm was abundant and eosinophilic; the cell nuclei were round and centered, the chromatin was uniform and coarse, and mitotic images were rare) (Figure 2C1,C2) were found in tumor parenchyma.

### 3.4. Immunohistochemical Methods

Most studies indicated that there were argyrophilic and argentophil particles in the carcinoid cells of SCTO, and thyroglobulin was present in thyroid follicular cells by immunohistochemical analysis. Carcinoid cells can express neuroendocrine markers CgA and Syn, as well as various peptide hormones, such as pancreatic polypeptide, gastrin, vasoactive intestinal peptide, YY polypeptide, etc. [28]. CK7 is usually expressed in the glandular epithelium, which is a non-specific indicator, so the expression of CK7 in carcinoid cells is uncertain. The thyroid tissue expresses specific indicators such as TTF-1, TG, TPO, etc., but not neuroendocrine indicators. Ki-67 is a marker of cell proliferation, whose positive expression is positively correlated with the malignant degree of the tumor [29]. Topoisomerase IIα and Ki-67 were found highly positive in a report of malignant tumor metastasis, which were considered as the characteristics of ovarian carcinoid tumor of “atypical carcinoid”, suggesting a poor prognosis [30].

Multiple groups have found that immunohistochemical tests for STCO generally exhibit at least one neuroendocrine marker [9], with the highest expression of Syn and CD56. Similarly, in 13 case reports over 5 years, we found that Syn (13/13), CgA (7/13), and CD56 (5/13) were the most highly expressed in carcinoid tissues. Ki-67 often showed low proliferative activity (2%); CK7 was positive (4/13); one raw of constipation patient was positive; one case of combined appendiceal carcinoid ovarian carcinoid tissues showed positive PAX8. Interestingly, PAX8 was negative for appendiceal carcinoid tissues. This suggests that the case is a primary carcinoid tumor of the ovary rather than a metastasis from an appendiceal carcinoid tumor [20]. In this case, the neuroendocrine markers CgA and Syn in the carcinoid cells were strongly positive (Figure 4D,E), and the specific indicator Tg of the thyroid was positive (Figure 4B). CK20 was negative in the carcinoid area, but positive in the colon gland area with teratomas (Figure 4C), with Ki-67 (5%+) low in expression (Figure 4F). Thus, in this case, the disease can be diagnosed as mature cystic teratoma of ovary with goitrous carcinoid and with a lower malignancy.

### 3.5. Operative Treatment

Operation is the primary therapeutic method of SCTO. Most primary ovarian carcinoid tumors develop slowly, and almost all thyroid carcinoid tumors are in clinical stage I with a favorable prognosis. Different therapeutic schedules should be adopted according to the age, stage, and reproductive requirements of patients. For the patients in clinical stage I, if the tumor is confined to the ovary, the young patient with fertility requirements may undergo unilateral salpingo-oophorectomy. The whole hysterectomy and bilateral salpingo-oophorectomy or cytoreductive surgery are feasible for the elderly patients without reproductive function. Omentectomy and para-aortic lymphadenectomy may also be required in patients with rare ovarian carcinoid mucin variation, as these tumors diffuse primarily through lymphatic vessels [31].

Of the 117 patients collected by Turla A et al. [12], 99% patients underwent tumor removal (unilateral/bilateral salpingo-oophorectomy/total hysterectomy). Three patients had metastatic disease at diagnosis and five patients underwent recurrence after radical surgery. Histology at disease recurrence concerned the thyroid component in two patients, the carcinoid component in two patients, and both histologies in one patient. Median disease-free survival and overall survival in this series were not attained. The remaining 112 had no recurrence at a mean follow-up of 2 years. Several studies have concluded that strumal carcinoids are the most common well-differentiated tumors/malignant neoplasms that exhibit little or no invasive clinical behavior. Regardless of the surgical approach, the prognosis is mostly good. Therefore, conservative surgery with individualized adjuvant therapy is recommended [32].

In this case, the tumor was confined to the left ovary, and the cyst tissue was completely removed, but the cyst was ruptured during the operation. The patient was considered to be stage IC 1 left ovarian mature cystic teratoma with goitrous carcinoid. The incidence rate of preoperative malignant tumor, in this case, was 36.0%, indicating that the risk of the malignant tumor was high. Therefore, the possibility of the malignant tumor should be considered before the operation. In this case, the cyst ruptured during the operation, leading to the spread of cystic fluid in the pelvic cavity and abdominal cavity. After the subsequent discussion of the case, we reflected on whether we should conduct an adequate evaluation of the tumor character before the operation and detailed family conversation prior to the deliberate decision on the surgical option. In addition, when the malignant tumor was found after the operation, it was recommended that the patient be readmitted for laparoscopic left adnexectomy in consideration of the low-grade malignant tumor, which was also combined with the patient’s young age, the confinement of the tumor to one ovary, as well as the rupture of the intact capsule during the operation. The patient and the patient’s family members finally considered follow-up and did not perform the further operation. However, a small percentage of patients with this disease may still recur due to a combination of thyroid and carcinoid components. Therefore, radical surgery is still required for patients with recurrence. Furthermore, based on the cases and data reported so far, the SCTO may occasionally metastasize [33], such as multiple metastases to the breast and bone [30]. In recent years, lymph node metastasis has also been reported [5]. Except for the special cases mentioned above, almost all the remaining cases have benign processes without metastasis. Therefore, it was concluded that this case could be followed up on the premise of closely following up on tumor indicators, endocrine function, breast, and other indicators.

## 4. Conclusions

Through this case report, we would like to indicate that specific imaging examinations, as well as serological tests for malignant tumors of the ovary, have shown low specificity and sensitivity in detecting this rare malignant tumor of the ovary. Moreover, most patients can have no clinical symptoms, which increases the difficulty of preoperative diagnosis. Abdominal CT and MRI can effectively assist doctors when ultrasound is unable to preliminarily judge the benign and malignant tumors, meanwhile, they can also be combined with the international ovarian tumor risk assessment model to conduct risk assessment. Although SCTO is difficult to make a clear diagnosis before operation, SCTO has the features of releasing YY peptide, endocrine dysfunction, steroid hormone secretion, etc., hence, when it comes to female patients with chronic habitual constipation, abdominal colic, diarrhea, abnormal menstruation, and crinosity, we should be alert to the occurrence of this rare malignant tumor of the ovary. For the choice of surgical approach for the tumor, because most of them show benign tumor behavior, conservative surgery can achieve the purpose of removing the tumor, while for patients with recurrence, radical surgery is still required.

## Figures and Tables

**Table 1:** Classification and clinical manifestation of ovarian strumal carcinoid.

Authors (Year)	Patients (N)	Clinical Manifestations	Mean Size	Classification	Immunohistochemical
Takatori E et al. (2012) [6]	6	abdominal mass (2) Constipation (1)	11	Trabecular	CgA+ Syn+ TG+ PYY+
Muller KE et al. (2014) [7]	14	Constipation (13)	9.9	Strumal (5) Trabecular (4) Strumal+Trabecular (5)	PYY++ (10)
Erdenebaatar C et al. (2015) [8]	20	Constipation (18)	9.7	Strumal (8) Trabecular (6) Strumal + Trabecular (5)	PYY++
Ge HJ et al. (2018) [9]	15	abdominal mass (3) colporrhagia (4) abdominal pain (1) Constipation (1)	8.8	Insular (3) Trabecular (1) Strumal (15) Mucinous (1)	Syn: + (14/15); CgA: + (10/15); CD56: + (9/15) Ki—67: <2%
Zhu R et al. (2018) [10]	17	abdominal pain (3)	8.2	Insular (4) Trabecular (4) Insular+Trabecular (2) Strumal (1) Mucinous (6)	NSE, Syn: +(17/17) CgA: + (10/17) Ki—67: ≤ 2% (17/17)
Yan F et al. (2021) [11]	4	abdominal mass (2)	8.9	Insular (1) Trabecular (1) Strumal (2)	CK, Syn, CgA and CD56: ++ TTF—1: − Ki67: 2%
Turla A et al. (2022) [12]	111	abdominal mass (41) abdominal pain (49) Constipation (54)	8	Trabecular (61) Insular + trabecular (44) Insular (5)	NEC, CgA, Syn, CD56: + (111) TTF—1: + (101) TG: +(98)

**Table 2:** Recent case reports of ovarian strumal carcinoids.

Authors (Year)	Patients (N)	Clinical Manifestations	Mean Size	Tumor Maker	Classification	Immunohistochemical	Concomitant Tumors
Noh HK et al. (2017) [13]	1	Constipation	6.4	Normal	Strumal	Syn, PAX8: + PYY+	Absent
Macháleková K et al. (2018) [14]	2	Absent	6.5 7.5	CA125:45 IU/mL	Trabecular Trabecular+Mucinous	Syn, CK7: + CgA: + (50%) Ki—67: < 2%	Mature cystic teratoma
Antovska VS et al. (2018) [15]	1	Absent	6	Normal	Insular+Trabecular	Syn, NSE, CD56: ++ CgA: + Ki—67: 10%	Mature cystic teratoma
Borghese M et al. (2019) [5]	1	Absent	4	Normal	Trabecular	Syn, NSE, CD56: + Tg:+/−, CDX2: +/− Ki—67: < 2%	Mature cystic teratoma /metastasis
Chai W et al. (2019) [16]	1	Abdominal mass	8.8	CA125: 768.10 IU/mL	Insular	Syn, CgA, NSE: +	Absent
Ishida M et al. (2019) [17]	1	Absent	5	Normal	Trabecular	Syn: ++	Absent
Mohammed SY et al. (2021) [18]	1	Abdominal pain Anorexia Frequent urination	24	CA125:105 IU/mL; CEA: 6.4 ng/mL	Insular+Trabecular	Syn, CgA: +	Mature cystic teratoma
Turla A et al. (2022) [12]	1	Abdominal pain Constipation	4	Normal	absent	Syn, CgA, CD56: + CK7: + Ki—67: 2%	Absent
Yuan Y et al. (2022) [19]	1	Absent	9	absent	Strumal	CD56, NSE, PSAP, CDX2: + AE1/AE3: +	Mature cystic teratoma
Upasana Baruah et al. (2022) [20]	1	Abdominal distension	15	CA125:944 IU/mL; CA199:944 ng/dL	Strumal	Syn, CD56, PAX8: + Ki—67 < 3%	Mature cystic teratoma /Appendix carcinoid
Cagino K et al. (2022) [21]	1	Abdominal pain Colporrhagia	10	absent	Insular	NCE, Syn, CgA: +	Mature cystic teratoma

## Data Availability

The data presented in this article are available on request from the corresponding author. They are not publicly available due to patients’ data protection regulations.
